# Molecular linkage between post-traumatic stress disorder and cognitive impairment: a targeted proteomics study of World Trade Center responders

**DOI:** 10.1038/s41398-020-00958-4

**Published:** 2020-08-04

**Authors:** Pei-Fen Kuan, Sean Clouston, Xiaohua Yang, Roman Kotov, Evelyn Bromet, Benjamin J. Luft

**Affiliations:** 1 Department of Applied Mathematics and Statistics, Stony Brook University, Stony Brook, NY USA; 2 Department of Family and Preventive Medicine, Stony Book University, Stony Brook, NY USA; 3 Department of Medicine, Stony Brook University, Stony Brook, NY USA; 4 Department of Psychiatry, Stony Book University, Stony Brook, NY USA

**Keywords:** Diagnostic markers, Molecular neuroscience

## Abstract

Existing work on proteomics has found common biomarkers that are altered in individuals with post-traumatic stress disorder (PTSD) and mild cognitive impairment (MCI). The current study expands our understanding of these biomarkers by profiling 276 plasma proteins with known involvement in neurobiological processes using the Olink Proseek Multiplex Platform in individuals with both PTSD and MCI compared to either disorder alone and with unaffected controls. Participants were World Trade Center (WTC) responders recruited through the Stony Brook WTC Health Program. PTSD and MCI were measured with the PTSD Checklist (PCL) and the Montreal Cognitive Assessment, respectively. Compared with unaffected controls, we identified 16 proteins associated with comorbid PTSD–MCI at *P* < 0.05 (six at FDR < 0.1), 20 proteins associated with PTSD only (two at FDR < 0.1), and 24 proteins associated with MCI only (one at FDR < 0.1), for a total of 50 proteins. The multiprotein composite score achieved AUCs of 0.84, 0.77, and 0.83 for PTSD–MCI, PTSD only, and MCI only versus unaffected controls, respectively. To our knowledge, the current study is the largest to profile a large set of proteins involved in neurobiological processes. The significant associations across the three case-group analyses suggest that shared biological mechanisms may be involved in the two disorders. If findings from the multiprotein composite score are replicated in independent samples, it has the potential to add a new tool to help classify both PTSD and MCI.

## Introduction

Studies of the long-term psychiatric and neurocognitive functioning of World Trade Center (WTC) responders during the two decades since September 11, 2001 have found high rates of impairment. The most prevalent psychiatric condition is post-traumatic stress disorder (PTSD), which is characterized by re-experiencing, avoidance, negative cognitions and mood, and arousal symptoms^1–3^. Nearly 20% of responders developed PTSD, and 10% continue to suffer from the disorder^1,4^. The most prevalent neurocognitive condition is mild cognitive impairment (MCI), which is characterized by declines in memory, learning, concentration, and decision-making that are not yet sufficient to cause functional limitations^5^. Critically, systematic reviews have identified consistent associations between PTSD and both neurocognitive dysfunction^6^ and dementia^7^ in cohorts of veterans and Holocaust survivors. In our WTC cohort, we observed a 2.67-fold increase in the incidence of MCI among responders with PTSD two decades after exposure^8^. Given this association, this paper uses proteomics analysis to undertake an in-depth characterization of the pathophysiology of MCI, PTSD, and their co-occurrence.

Proteomics is a promising strategy for characterizing the biological signatures of disorders that has been facilitated by the emergence of high-throughput technologies^9^. Proteins execute functions within cells and communication between them, and thus are potentially involved in pathological processes underpinning PTSD and MCI. Proteomics, therefore, aims to capture the dynamics of protein expression and detail their interactions within a cell^10^, an important process when trying to elucidate cellular adaptation to environmental signals and cellular aspects of disease processes^11^. Proteomics offers a different level of understanding of these processes compared with genomics and transcriptomics because proteins undergo alternative post-translational modification (e.g., phosphorylation) essential for protein function; as a result, information from a single gene can encode different protein species^10^ and form protein complexes that determine function^11^.

Existing work on proteomics has identified biomarkers that are altered both in individuals with PTSD and with MCI. For example, PTSD has been linked to alterations of serum proteins such as glial fibrillary acidic protein (GFAP), vascular endothelial growth factor (VEGF)^12^, β-amyloid^13^, and C-reactive protein (CRP)^14^. Similarly, MCI was associated with changes to VEGF, CRP, and cortistatin (CORT), among others^15^. Co-occurring PTSD and MCI was examined in only one molecular study of a mouse model that found that the loss of FMN2 gene was associated with both PTSD-like phenotypes (i.e., fear extinction) and age-accelerated memory impairment^16^. However, no studies to our knowledge have examined the extent to which protein signatures for PTSD, MCI, and their comorbidity differ in vivo in humans. This is important because of known interspecies variability and differences in proteomics^17^.

This study aims to fill the gap in molecular studies of PTSD and MCI by profiling a large set of proteins (*k* = 276) with known involvement in related processes to determine whether markers of neurodevelopmental processes, cellular regulation, immunological function, cardiovascular disease, inflammatory processes, and neurological diseases are linked to PTSD and MCI by comparing patients with PTSD, MCI, and comorbid PTSD–MCI with unaffected controls^18–20^. We hypothesized that alterations in these processes reflect a combination of proteomic profiles that are observed in PTSD, MCI, and comorbid PTSD–MCI but not in unaffected individuals. Second, we constructed multiprotein composite scores and examined their associations with PTSD and MCI symptom severity.

## Methods

### Participants

Participants were recruited through the Stony Brook WTC Health Program^21^. This study was approved by the Stony Brook University IRB. Written informed consent was obtained. The analysis focused on a subsample of male responders who completed their annual monitoring visit in 2019. We studied only male responders because <10% of the Stony Brook cohort is female, and women show notably different protein expression patterns from men^22^. Responders with a history of medical or neurodegenerative conditions, brain tumors, cancers, or cerebrovascular conditions were ineligible for the study.

### Clinical measures and classification

Probable PTSD was measured with the Posttraumatic Stress Disorder Checklist-Specific Version (PCL-17)^23^, a 17-item self-report questionnaire modified to assess the severity of WTC-related DSM-IV PTSD symptoms over the past month on a scale of 1 (never bothered by) to 5 (extremely bothered by) (Cronbach α = 0.96). Probable PTSD was operationalized by a PCL total score >44. The unaffected sample was asymptomatic (PCL score <22).

MCI was measured using the Montreal Cognitive Assessment (MoCA), a widely used objective multidomain test^24^. A conservative cutoff of <22 was applied to reduce misclassification. Normal cognitive functioning was defined as MoCA >26 consistent with testing guidelines^25^. Unaffected controls (PCL <22 and MoCA >26) were subject to an additional medical record review to rule out responders with a clinical history of PTSD and related disorders.

The final sample (*N* = 181) included 34 responders with comorbid PTSD–MCI, 39 with PTSD only, 27 with MCI only, and 81 unaffected controls.

### Proteomics profiling

Protein expression of plasma was profiled using the Olink Proseek Multiplex Platform. The Olink multiplex immunoassay was designed to provide an ultrasensitive, reproducible, and highly multiplexed method for measuring protein expression. The measurement was based on state-of-the-art Proximity Extension Assay (PEA) technology^26^. More details are available online (https://www.olink.com). Three commercial Olink panels were profiled for each participant included in the Neurology, Neuro Exploratory and Cardiovascular II (CVII) panels. Thus, 276 proteins (92 proteins per panel) were targeted involving a range of processes indicative of a range of neurological diseases, cellular regulation, immunology, cardiovascular, inflammatory, development, and metabolism.

### Proteomics data preprocessing

A number of internal and external controls were added to the plasma samples for quality control to monitor protein–antibody reactions, the DNA extension step, and detection quality of the qPCR in order to estimate the background signal and to calculate the limit of detection (LOD) for Olink panels. Proteins below LOD were imputed with LOD^27^. Protein concentration was represented in arbitrary units on a log_2_ scale and termed Normalized Protein eXpression (NPX), i.e., a one NPX difference means a doubling of protein concentration. The NPX value represented a relative quantification so that the data for a specific protein can be compared across different samples. Reference samples run on plates from different batches were included for batch-effect correction. The adjustment factor at protein level for each batch was calculated as median NPX of the bridging samples and subtracted from the NPX values of each sample. Batch-corrected log- transformed NPX was used in subsequent analyses (termed normalized NPX). We compared the reproducibility of the bridging samples using Pearson correlation. Supplementary Figure 1 shows the high reproducibility of the Olink panels across six representative sets of technical duplicates, with a mean correlation *r* = 0.97.

### Differential proteomics analysis

To assess associations of PTSD and MCI with protein regulation, differential analyses were carried out using a linear model with normalized NPX as the dependent and case/control as independent variables, adjusting for age and race, on a subset of (a) 34 PTSD–MCI cases versus 81 unaffected controls, (b) 39 PTSD-only cases versus 81 unaffected controls, and (c) 27 MCI-only cases versus 81 exposed controls. Statistically significant proteins were identified at *P* < 0.05, as well as at false discovery rate (FDR) < 0.1 within each panel^28^. To assess the consistency of the findings, a Monte-Carlo experiment was conducted by randomly partitioning the data into 50% discovery and 50% replication subsample. We considered replicated proteins in which both the discovery and replication subsamples were significant at *P* < 0.10, and had effect sizes in the same direction. The random partitioning was repeated 100 times, and the number of times the proteins were replicated was recorded. The correlation between the estimated beta coefficients of all proteins for case/control status across the three subset analyses was assessed using Pearson correlation coefficients. The overlap between the top proteins identified from each subset analysis was compared via a Venn diagram. The top proteins identified from this study were compared with recent omics studies of PTSD and Alzheimer’s disease (AD).

### Disease-burden analysis

Among the proteins identified at FDR < 0.1 from the PTSD–MCI subset analyses, three competing models were fitted to ascertain which of the following models best fit the protein- regulatory pattern: *H1*, the protein expression of PTSD-only subgroup was intermediary between PTSD–MCI and control (i.e., Control < PTSD only < PTSD–MCI or Control > PTSD only > PTSD–MCI), *H2*, the protein expression of the PTSD-only subgroup was similar to PTSD–MCI subgroup (i.e., Control ≠ PTSD only = PTSD–MCI), or *H3*, the protein expression of PTSD-only subgroup was similar to the unaffected controls (i.e., Control = PTSD only ≠ PTSD–MCI). For model *H1*, a linear model was fitted to the subgroup defined by 1 = control, 2 = PTSD only, and 3 = PTSD–MCI as an ordinal predictor. For model *H2*, a linear model was fitted to the subgroup defined by 0 = control, 1 = PTSD only, or PTSD–MCI as a binary predictor. For model *H3*, a linear model was fitted to the subgroup defined by 0 = control or PTSD only, 1 = PTSD–MCI as a binary predictor. All models were adjusted for age and race. The Bayesian Information Criterion (BIC) score was computed, and the model that corresponded to the smallest BIC score was selected as the best-fitting model. Analyses were repeated by replacing PTSD-only subgroup with MCI-only subgroup. Proteins that identified model *H1* as the best-fitting model can be regarded as candidate biomarkers for disease burden characterized by co-occurrence of PTSD–MCI.

### Multiprotein composite score

To evaluate the utility of proteomics in classifying cases and controls, we applied the elastic net algorithm^29^. For each case/control subset, the top-ranking proteins by *P* values from the differential expression analysis were used as candidate feature sets. Leave-one-out (LOO) cross-validation prediction was used to evaluate model performance, i.e., the model was trained on N-1 samples, and used to predict the score in the left-out test sample, and the process was cycled through N samples. Within each training set, the optimal tuning parameters were determined via a fivefold cross-validation. The area under the ROC curve (AUC) was used as a metric for performance evaluation. Pearson correlation was calculated to estimate the association between the multiprotein composite scores and PTSD and MCI symptom-severity score.

## Results

### Participant characteristics

The overall average age was 55.1 (SD = 7.78), and the mean ages of the four groups were similar. The majority of the sample was Caucasian, and no significant racial/ethnic differences among cases and controls were observed (Table 1).

**Table 1:** Clinical characteristics of study samples.

	PTSD–MCI, *N* = 34	PTSD only, *N* = 39	MCI only, *N* = 27	Control, *N* = 81	*P* value
*Age*					
Mean (SD)	56.67 (8.15)	56.31 (8.82)	56.52 (6.36)	53.40 (7.30)	0.067
*Race,* N (%)					
Caucasian	25 (73.5)	36 (92.3)	22 (81.5)	70 (86.4)	0.147
Other	9 (26.5)	3 (7.7)	5 (18.5)	11 (13.6)	

The *P* values were computed from one-way ANOVA (for age) and chi-squared test (for race).

### Differential protein analysis associated with PTSD and MCI

**Fig. 1:**
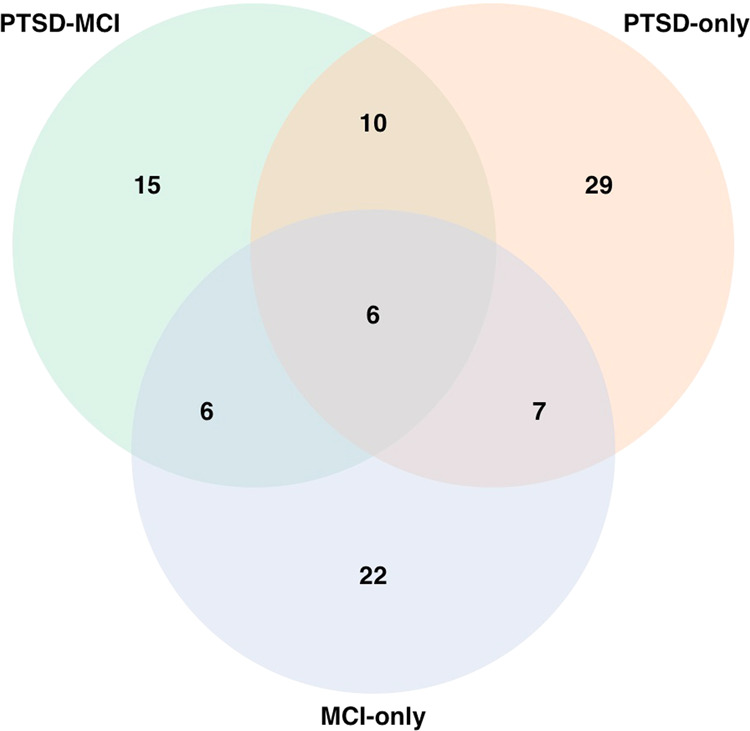
Overlap between the top proteins at *P* < 0.05 from the three subset analyses. PTSD only (24 proteins), MCI only (20 proteins), and PTSD–MCI (16 proteins).

Subset analysis of comorbid PTSD–MCI case group versus controls identified 16 Olink proteins at *P* < 0.05, of which six attained FDR < 0.1. Eleven of the original 16 proteins were upregulated in cases. The six proteins significant at FDR < 0.1 were NCAN, BCAN, CTSS, MSR1, MDGA1, and CPA2; all six proteins were replicated >50% times in the Monte-Carlo experiment. On the other hand, subset analysis of PTSD-only cases versus controls identified 24 proteins at *P* < 0.05, of which two attained FDR < 0.1. In total, 22 out of these 24 proteins were upregulated in cases. The two proteins significant at FDR < 0.1 were CD302 and FLRT2; both were replicated >70% times in the Monte-Carlo experiment. Finally, subset analyses of MCI-only cases versus controls identified 20 proteins at *P* < 0.05, of which only one attained FDR < 0.1. Seven out of these 20 proteins were upregulated in cases. The protein significant at FDR < 0.1 was PVR, which was replicated >80% times in the Monte-Carlo experiment. Altogether, 50 unique proteins were obtained from the combined lists in subset analyses (Table 2). Several identified proteins had been previously implicated in other omics studies of PTSD and AD. Additional details on comparison of these proteins with recent omics studies of PTSD and AD were provided in Supplementary Text and Supplementary Tables 2–4. The Venn diagram comparing the overlap between the top proteins in subset analyses (Fig. 1) suggested that CTSS was the only common protein identified by all subset analyses at *P* < 0.05, whereas EFNA4 was in common between PTSD–MCI and PTSD-only analyses; BCAN, MDGA1, CPA2, and EPHA10 were in common between PTSD–MCI and MCI-only analyses; PVR, CD200, and ATP6V1F were in common between PTSD-only and MCI-only analyses.

**Table 2:** List of proteins differentially expressed at *P* < 0.05 from the subset analyses.

Name	Description	Up/ down in PTSD–MCI	*P* value in PTSD–MCI	AUC in PTSD and MCI	Up/ down in PTSD only	*P* value in PTSD only	AUC in PTSD only	Up/ down in MCI only	*P* value in MCI only	AUC in MCI-only
Neurocan core protein (NCAN)	Modulates neuronal adhesion and neurite growth, implicated BPD, SCZ^47^, and AD^49^	Down	<0.0001*	0.7745	Down	0.1735	0.6315	Down	0.0842	0.599
Brevican core protein (BCAN)	Implicated in central nervous system, neuronal synapse plasticity, and extracellular matrix of the brain, implicated in AD^39^	Down	0.0008*	0.7284	Down	0.0718	0.6306	Down	0.0083	0.7055
Cathepsin S (CTSS)	Expressed by antigen-presenting cells, play a role in immune responses. Cathepsins are implicated in AD^35,36^, SCZ^38^, and PTSD^33^	Up	0.0014*	0.6659	Up	0.0200	0.6173	Up	0.0232	0.6351
Macrophage scavenger receptor types I and II (MSR1)	Implicated in macrophage-associated physiological and pathological processes, including AD^52^, MDD^53^, SCZ and BPD^54^, and PTSD^42,43^	Up	0.0023*	0.7073	Up	0.0820	0.6277	Up	0.9440	0.5542
MAM domain-containing glycosylphosphatidylinositol anchor protein 1 (MDGA1)	Needed for the radial migration of cortical neurons in the neocortex, and implicated in SCZ^55^, autism^56^, and AD^39^	Up	0.0031*	0.6434	Up	0.0679	0.5818	Up	0.0266	0.6237
Carboxypeptidase A2 (CPA2)	Carboxypeptidase enzymes are implicated in AD^57^ and SCZ^58^	Up	0.0031*	0.6489	Up	0.2779	0.5476	Up	0.0161	0.6621
Lectin-like oxidized LDL receptor 1 (LOX-1)	Main receptor for oxidized LDL on a number of cells, including endothelial cells and macrophages, implicated in AD^59^	Up	0.0079	0.6797	Up	0.3093	0.5606	Up	0.0976	0.5684
P-selectin glycoprotein ligand 1 (PSGL-1)	Involved in the recruitment of white blood cells into inflamed tissue, regulates immune checkpoint	Up	0.0178	0.6285	Up	0.2119	0.5606	Up	0.0628	0.6127
Neurexophilin-1 (NXPH1)	Binds to alpha-neurexins and promotes adhesion between dendrites and axons. Implicated in AD^39^ and SCZ^60^	Down	0.0217	0.6503	Down	0.2043	0.5761	Down	0.2474	0.599
Interleukin-1 receptor antagonist protein (IL-1ra)	A cytokine involved in immune and inflammatory responses. Upregulated in SCZ^61^, PTSD^62^, and AD^63^	Up	0.0241	0.6511	Up	0.2099	0.5932	Up	0.4326	0.5743
Fibroblast growth factor receptor 2 (FGFR2)	Involved in neurogenesis and neurodegeneration, associated with BPD^64^ and SCZ^65^	Up	0.0259	0.6126	Up	0.2396	0.5616	Down	0.8937	0.5176
Ephrin type-A receptor 10 (EPHA10)	Involved in mobility in neuronal and epithelial cells and memory formation, Eph/ephrin system is implicated in AD^39^	Down	0.0265	0.5881	Down	0.5581	0.5334	Down	0.0167	0.7156
Gastrotropin (GT)	Involved in fatty acid uptake, transport, and metabolism	Down	0.0319	0.6046	Down	0.1769	0.5986	Down	0.2847	0.5871
Tumor necrosis factor receptor superfamily member 27 (EDA2R)	Mediates cell–cell signaling, associated with age and developmental brain disorders including autism^66^	Up	0.0346	0.7015	Up	0.6121	0.5973	Up	0.2194	0.6374
Ephrin-A4 (EFNA4)	Implicated in mediating developmental events in the nervous system and autism^67^	Up	0.0385	0.6398	Up	0.0050	0.6572	Up	0.3643	0.5706
Matrix metalloproteinase-12 (MMP-12)	Involved in the breakdown of extracellular matrix in normal physiological processes. MMPs are implicated in memory and neuropsychiatric disorders^68^ and AD^69^, potential drug target for SCZ^70^	Up	0.0398	0.6837	Up	0.2194	0.5986	Up	0.3234	0.6173
CD302 antigen (CD302)	Implicated in endocytosis, phagocytosis, cell adhesion, and migration	Up	0.0872	0.6481	Up	0.0006*	0.6727	Up	0.1721	0.5889
Leucine-rich repeat transmembrane protein (FLRT2)	Functions in cell–cell adhesion, cell migration (cortical neurons), and axon guidance, possibly implicated in SCZ^71^	Up	0.0762	0.6151	Up	0.0010*	0.6986	Up	0.0669	0.6255
Poliovirus receptor (PVR)	Implicated in the immune response, including mediating NK cell adhesion and triggering its effector functions^72^, BPD, SCZ^73^, and AD^74^	Up	0.0714	0.5813	Up	0.0038	0.648	Up	0.0005*	0.7558
Interferon lambda-1 (IFNL1)	A cytokine involved in host defense and immunity	Up	0.4141	0.5781	Up	0.0084	0.6632	Up	0.0912	0.6607
PDGF-R-alpha (PDGFRA)	Involves in cell proliferation	Up	0.5801	0.5403	Up	0.0105	0.6261	Up	0.0528	0.615
Calcineurin subunit B type 1 (PPP3R1)	SNP rs1868402 in the PPP3R1 gene is strongly correlated with rapid progress of AD^75^	Up	0.1058	0.6049	Up	0.0107	0.6527	Up	0.0987	0.6017
Beta-defensin 4 A (DEFB4A)	An antibiotic peptide that is locally regulated by inflammation	Up	0.2509	0.5857	Up	0.0137	0.6477	Down	0.4502	0.54
Tumor necrosis factor receptor superfamily member 21 (TNFRSF21)	Involved in neurodegeneration in the brain that causes AD^76^ and PTSD^43^	Up	0.1480	0.614	Up	0.0142	0.6233	Up	0.2920	0.5775
Pregnancy-specific beta-1-glycoprotein 1 (PSG1)	Implicated in immune response in the fetus	Up	0.3229	0.6619	Up	0.0170	0.6834	Up	0.1915	0.6461
Disintegrin and metalloproteinase domain-containing protein 22 (ADAM 22)	Implicated in central nervous system development and myelination in the peripheral nervous system	Down	0.8172	0.5007	Up	0.0244	0.6084	Up	0.1418	0.5903
Phosphoethanolamine/phosphocholine phosphatase (PHOSPHO1)	Involved in bone and cartilage matrix mineralization	Up	0.0712	0.618	Up	0.0285	0.6119	Up	0.7173	0.5793
Cathepsin C (CTSC)	A central coordinator for activation of serine proteases in immune/inflammatory cells. Cathepsins are implicated in AD^77^ and SCZ^78^	Up	0.2085	0.5864	Up	0.0287	0.6328	Up	0.1172	0.6388
OX-2 membrane glycoprotein (CD200)	May regulate myeloid cell activity and provide an inhibitory signal for the macrophage lineage in multiple tissues. Implicated in SCZ, BPD^79^, AD^39,46^, and PTSD^43,45^	Up	0.3739	0.553	Up	0.0292	0.6204	Up	0.0212	0.6822
Epithelial discoidin domain-containing receptor 1 (DDR1)	Involved in the regulation of cell growth, differentiation, and metabolism. May contribute to AD via reduced degradation of amyloid beta^80^. A susceptible gene for SCZ^81^	Up	0.0902	0.5737	Up	0.0296	0.5916	Up	0.9415	0.5332
Cardiotrophin-1 (CTF1)	A cytokine associated with the pathophysiology of heart diseases. Implicated in AD^39^	Down	0.8895	0.5763	Down	0.0297	0.6119	Down	0.2227	0.5949
Secreted frizzled-related protein 3 (sFRP-3)	An inhibitor of cell growth and differentiation, modulates WNT signaling, and regulates antidepressant responses^82^	Up	0.1960	0.529	Up	0.0323	0.6157	Down	0.6001	0.5281
Serine/threonine-protein kinase receptor R3 (SKR3)	Expressed in neuron, a regulator of normal blood vessel development	Up	0.0767	0.6416	Up	0.0329	0.6303	Up	0.6414	0.5382
C–C motif chemokine 27 (CCL27)	A cytokine involved in immunoregulatory and inflammatory processes. Implicated in AD^83^	Down	0.3241	0.5385	Up	0.0410	0.5904	Up	0.4513	0.5519
V-type proton ATPase subunit F (ATP6V1F)	Involved in regulation of luminal or extracellular acidification, a crucial process for the normal physiological function of several organs, implicated in PTSD^42,43^	Down	0.0613	0.6245	Down	0.0433	0.6486	Down	0.0405	0.6795
Brorin (VWC2)	Involves in neural development, implicated in AD^39^	Up	0.3971	0.5654	Up	0.0444	0.6572	Up	0.2008	0.6187
Desmoglein-3 (DSG3)	Implicated in autoimmune disease^84^	Up	0.2187	0.6137	Up	0.0452	0.6081	Up	0.2013	0.5853
DRAXIN	Plays an important role in neural development	Up	0.3633	0.5632	Up	0.0465	0.5996	Up	0.4593	0.5222
Cadherin-15 (CDH15)	May contribute to the sorting of heterogeneous cell types. Implicated in PTSD^85^, cognitive impairment^86^	Down	0.9755	0.5105	Down	0.3462	0.5609	Down	0.0024	0.7165
NEDD8-conjugating enzyme UBE2F (UBE2F)	Implicated in stress response	Up	0.1836	0.5203	Down	0.2715	0.6208	Down	0.0029	0.7572
SPARC-related modular calcium-binding protein 2 (SMOC2)	Promotes matrix assembly, stimulates endothelial cell proliferation and migration, implicated in AD^87^	Down	0.1552	0.5686	Down	0.0812	0.5929	Down	0.0056	0.6836
Neuropilin-2 (NRP2)	May play a role in neuron development and axon guidance. Implicated in AD^88^, PTSD^89^, andMDD^90^	Up	0.7498	0.5675	Up	0.0928	0.5859	Up	0.0063	0.6941
Fibroblast growth factor 23 (FGF-23)	Regulator of inflammatory cytokine gene expression.	Up	0.4603	0.5054	Up	0.3346	0.5935	Down	0.0071	0.6557
Vascular endothelial growth factor D (VEGFD)	Involves in angiogenesis, lymphangiogenesis, and endothelial cell growth. Implicated in SCZ^91^ and AD^92^	Down	0.2748	0.5428	Down	0.5774	0.5356	Down	0.0090	0.6918
Tubulin polymerization-promoting protein family member 3 (TPPP3)	May play a role in cell proliferation and mitosis. Implicated in AD^93^ and SCZ^94^.	Up	0.8834	0.504	Down	0.4315	0.5239	Down	0.0181	0.6511
Latexin (LXN)	Implicated in PTSD^43,45^	Up	0.1175	0.5483	Down	0.4287	0.5878	Down	0.0205	0.7147
N-Myc downstream regulated 1 (NDRG1)	Involved in hormone responses, cell growth, differentiation, and aging. Implicated in PTSD^95^, AD^96^, MDD, and SCZ^97^	Down	0.9984	0.5022	Up	0.1899	0.5419	Down	0.0310	0.6667
Low-affinity immunoglobulin gamma Fc region receptor II-b (IgG Fc receptor II-b)	Regulates B-cell activation, implicated in AD^98^	Down	0.1971	0.5236	Down	0.2643	0.5486	Down	0.0408	0.6374
Transmembrane glycoprotein NMB (GPNMB)	Expressed in melanocytes, osteoclasts, osteoblasts, and dendritic cells, increased expression in AD^39^	Up	0.0842	0.6213	Up	0.2879	0.5926	Up	0.0450	0.6232
Interleukin-15 (IL15)	Involves in innate and adaptive immunity. Implicated in PTSD^99^, AD^100^, and SCZ^9^	Down	0.3284	0.5679	Up	0.6530	0.5299	Down	0.0479	0.6187

*SCZ* schizophrenia, *BPD* bipolar disorder, *AD* Alzheimer’s disease, *MDD* major depression, *PTSD* post-traumatic stress disorder.

*Statistically significant after accounting for the false discovery rate (FDR < 0.10).

**Fig. 2:**
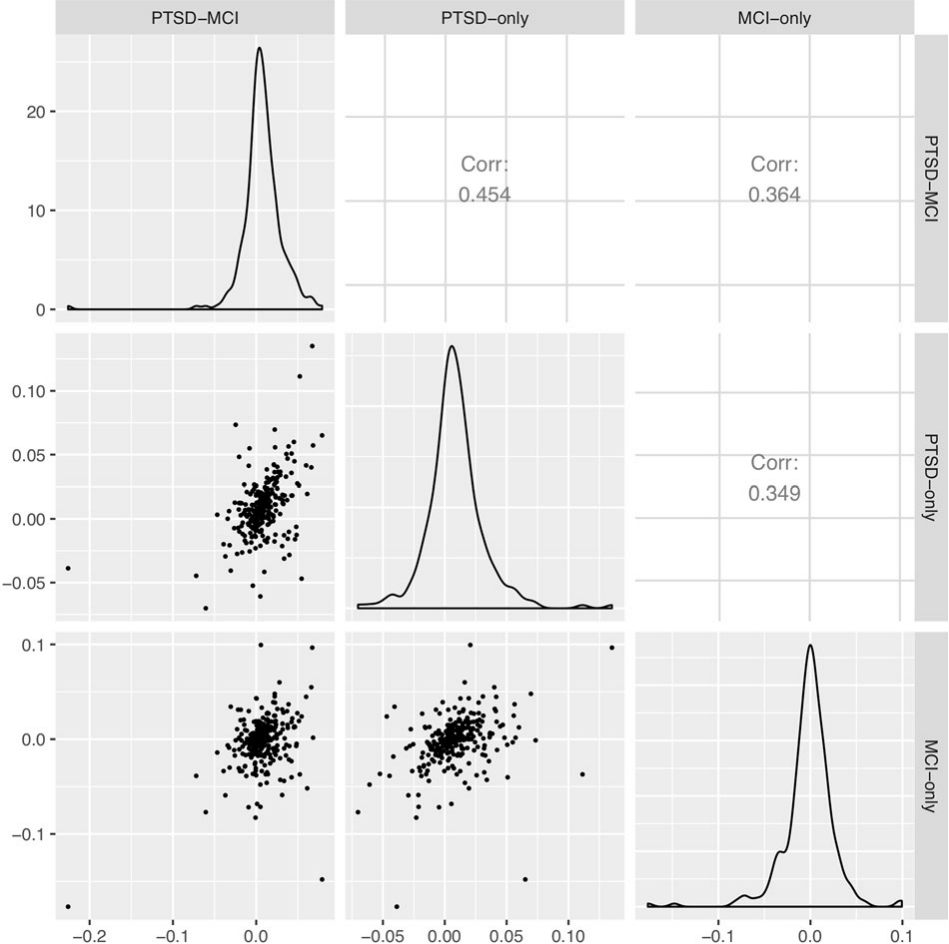
Pairwise correlations between the estimated beta coefficients for case/control across the three subset analyses, namely PTSD-MCI vs control, PTSDonlyvs control and MCI-only vs control. The lower triangular panel shows the scatter plots, the upper triangular panel shows the corresponding Pearsoncorrelation coefficients, the diagonal panel shows the distributions of the estimated beta coefficients.

Among 50 unique proteins identified above, 39/50 showed consistent sign/direction in the estimated beta coefficients across the three subset analyses. The remaining 11 proteins were not among the proteins shared by any two subset comparisons. Across all 276 proteins examined in these analyses, the estimated beta coefficients for PTSD only versus controls and MCI only versus controls were moderately correlated (*r* = 0.345, *P* < 0.05) as shown in Fig. 2, suggesting that shared biological mechanisms may be involved in the two disorders.

### PTSD–MCI-associated proteins linked to disease burden

Among the six proteins significant at FDR < 0.1 in the PTSD–MCI versus healthy control analysis shown in Table 2, BCAN and NCAN showed monotonically decreasing protein expression patterns, whereas for PTSD only versus PTSD–MCI, CTSS, MSR1, MDGA1, and CPA2 showed monotonically increasing protein expression patterns (Supplementary Fig. 2). The BIC scores are reported in Supplementary Table 5. All the proteins (except NCAN) achieved the lowest BIC scores in the *H1* model (i.e., the protein expression of the PTSD-only subgroup was intermediary between PTSD–MCI and control). The BIC scores of *H1* and *H3* models (i.e., control = PTSD only ≠ PTSD–MCI) of NCAN were comparable, indicating that both models fit NCAN equally well, and suggesting that these proteins are associated with disease burden of co-occurring PTSD and MCI compared with PTSD only. On the other hand, only for NCAN, *H1* was the best model. The protein expression of BCAN, CTSS, MDGA1, and CPA2 indicated that the MCI-only subgroup was similar to PTSD–MCI since the BIC scores for *H2* model (i.e., control ≠ MCI only = PTSD–MCI) were the lowest, whereas for MSR1, the MCI-only subgroup was similar to controls. These results suggest that the dysregulations of BCAN, CTSS, MDGA1, and CPA2 were most strongly associated with MCI.

### Multiprotein composite score

The leave-one-out (LOO) cross-validation achieved an AUC = 0.84 in PTSD–MCI classification (Table 3) using the top 37 proteins associated with PTSD–MCI at *P* < 0.1 listed in Supplementary Table 6 as candidate features. The AUC was lower at 0.81 using the 16 proteins associated with PTSD–MCI (*P* < 0.05). Similarly, the LOO cross-validation achieved AUC = 0.83 and 0.84 in MCI-only classification using the 20 and 41 MCI-only associated proteins (*P* < 0.05 and *P* < 0.1), respectively. However, the LOO cross-validation only achieved AUC 0.77 in PTSD-only classification (Table 3) using the 52 PTSD-only associated proteins at *P* < 0.1 listed in Supplementary Table 6. The AUC was lower (0.68) using 24 PTSD-only associated proteins at *P* < 0.05 (Supplementary Table 7). In all three classification models, using all 276 proteins as candidate features achieved a lower AUC, suggesting that adding in other protein signals may induce noise (Supplementary Table 7). Taken together, the results from multiprotein composite scores indicated that the panel of proteins included in this study had larger discriminative power for MCI compared with PTSD.

**Table 3:** Leave-one-out cross-validation prediction performance on models trained on subsets of (a) PTSD–MCI, (b) PTSD only, and (c) MCI only versus controls.

Classification	Candidate feature set	AUC	Correlation with PCL	Correlation with MoCA score
PTSD–MCI versus control	37 PTSD–MCI-associated Olink proteins at *P* < 0.1 from Supplementary Table 6	0.84	0.57 (*P* < 0.001)	−0.54 (*P* < 0.001)
PTSD only versus control	52 PTSD-only associated proteins at *P* < 0.1 from Supplementary Table 6	0.77	0.36 (*P* < 0.001)	−0.12 (*P* = 0.18)
MCI only versus control	41 MCI-only associated proteins at *P* < 0.1 from Supplementary Table 6	0.83	0.24 (*P* = 0.01)	−0.52 (*P* < 0.001)

## Discussion

Prior studies have shown that chronic PTSD in the responders to the World Trade Center disaster is associated with systemic and neuropsychiatric conditions including MCI^30,31^. Furthermore, in some instances, we demonstrated that not only was there an association, but that PTSD helps to mediate the development and chronicity of these conditions, and may be linked to possible early dementia^32^. The current study was the largest study to evaluate the molecular link between PTSD and MCI in the same cohort. It profiled a large set of proteins involved in a number of neurobiological processes, neurological diseases, cellular regulation, immunology, cardiovascular, inflammatory, development, and metabolism. In this study, we systematically assessed changes in the proteome of WTC responders suffering from PTSD with and without comorbid MCI nearly two decades after the traumatic event, in order to identify biomarkers that could inform us the biologic changes in our patients as well as the nature of the relationship between these conditions. We found that both MCI and PTSD were associated with serologic proteinopathy. The results also suggested that comorbid PTSD–MCI was likely a more severe form of PTSD rather than a separate condition. Last, we found that protein dysregulation was more systematically associated with MCI. As such, the multiprotein composite score provided us with a novel method to characterize and monitor patients with both MCI and PTSD and, if confirmed in independent studies, may ultimately give us insights into potential novel therapeutic interventions.

We identified 16 proteins associated with PTSD–MCI at p < 0.05 (six at FDR < 0.1), 20 proteins associated with PTSD only (two at FDR < 0.1), and 24 proteins associated with MCI only (one at FDR < 0.1), resulting in a total of 50 unique proteins from the combined lists. It is important to note that protein expression in the blood does not represent protein production in any specific tissue, per se, but rather proteins secreted into the blood from multiple organs and tissues. This is in contrast to gene expression analysis that is derived from a specific tissue. Nonetheless, although overall comparison with recent omics studies in AD showed that most of the top genes identified in these studies did not overlap with our targeted panel of 276 proteins as described in Supplementary Text, there were some that did as described below. Among these 50 proteins, only Cathepsin S (CTSS) was in common across the three subset analyses. Our analyses identified positive associations across the three subset analyses (*r* = 0.35–0.45), suggesting shared biological mechanisms across these two phenotypes. Notably, the gene encoding Cathepsin S (CTSS) had been found to be upregulated in the discovery cohort of Dean Hammamieh^33^, and plays an important role in antigen presentation and immune responses^34^. Single-nucleotide polymorphisms (SNP) that map to the CTSS gene have been found to be associated with late-onset Alzheimer’s disease (AD)^35^. Other members of the Cathepsin family have also been shown to be implicated in AD (Cathepsins B and D)^36,37^ and SCZ (Cathepsin K)^38^. On the other hand, MAM domain-containing glycosylphosphatidylinositol anchor protein 1 (MDGA1) and ephrin type-A receptor 10 (EPHA10), which were identified in both the PTSD-only and MCI-only analyses, have been found to be associated with pathologic and clinical diagnoses of AD in the transcriptomes of postmortem brain^39^. MDGA1 is implicated in the radial migration of cortical neurons of the neocortex^40^, whereas EPHA10 is involved in mobility in neuronal and epithelial cells and memory formation^41^. Similarly, V-type proton ATPase subunit F (ATP6V1F) and OX-2 membrane glycoprotein (CD200), which were identified in both the PTSD-only and MCI-only analyses, have been found to be differentially expressed in the transcriptomes of peripheral blood cells of patients with PTSD^33,42,43^. Based on the transcriptome mega-analysis results of Breen Tylee^43^ (DE genes at *P* < 0.05 for each trauma-specific case–control cohort as evident in Supplementary Table 2 of Breen study), ATP6V1F and CD200 showed consistent effect-size direction in transcriptomic regulation compared with the proteomics results in our data. Specifically, ATP6V1F was downregulated in the gene expression of emergency-department trauma survivors^42^, consistent with the protein expression in our data. In addition, loss of function of ATP6V1F has been shown to be a potential enhancer of tau toxicity, a hallmark of AD^44^. Yet, CD200 was upregulated in childhood trauma and interpersonal trauma subgroups^45^, consistent with our proteomics data. CD200 expression was shown to be downregulated in the hippocampus and inferior temporal gyrus of AD patients^46^. The authors further showed that lower expression of CD200 receptor was observed in microglia compared with blood-derived macrophages. Thus, we hypothesized that the upregulation of CD200 in plasma samples of our study could be a consequence of cell migration to blood through the blood–brain barrier.

The top two proteins, namely neurocan (NCAN) and brevican (BCAN) core proteins, identified from analyses of PTSD–MCI versus controls showed monotonically decreasing protein expression patterns across the PTSD-only and MCI-only subgroups, suggesting that these proteins are candidate biomarkers for disease burden characterized by co-occurrence of PTSD and MCI. Genetic variation in NCAN has been shown to be a common risk factor for bipolar disorder and schizophrenia^47^, as well as in MCI^48^. In addition, NCAN and BCAN are members of the chondroitin sulfate proteoglycan (CSPG) protein families, and CSPGs are implicated in neurodegenerative diseases^49^. Specifically, CSPGs have been shown to accumulate in senile plaques in brains of patients with AD^49^, potentially suggesting that fewer CSPGs will penetrate into the blood in AD. Together with the previous epidemiologic findings that PTSD is associated with long-term cognitive decline^30,50^, this suggests that NCAN and BCAN may constitute novel biomarkers contributing to processes by which PTSD affects cognitive functioning.

The multiprotein composite score based on top PTSD–MCI and MCI-only associated proteins achieved a high accuracy (AUC = 0.84) in PTSD–MCI and MCI-only classification, respectively. On the other hand, the multiprotein composite score based on top PTSD-only associated proteins achieved AUC = 0.77 in PTSD-only classification. These results suggested that the proteins included in this study have a larger discriminative power for MCI compared with PTSD. We also found a robust association between the composite score, PTSD, and CI symptom severity. This suggested that the current multiprotein composite score may be further refined into a useful index that aids in classification.

### Strengths and limitations

This study has several strengths, including a large-scale high-precision multiplexed proteomic analysis of a large number of neurological, inflammatory, and immune-related proteins using validated panels, and a common trauma in all participants including controls. Nonetheless, our findings must be considered in the context of several limitations. First, our study is cross-sectional, which can establish concurrent associations between protein expression, PTSD, and MCI. However, the direction of the associations cannot be determined. Longitudinal studies of linkages between change in symptom severity and change in protein expression are needed to determine the direction of the effects we observed. Second, potential confounders, such as the level of trauma exposure and comorbid medical conditions, were not considered. Third, the multiprotein composite score was constructed based on the proteins identified from the same study samples. Although we used a LOO cross-validation prediction scheme to reduce the bias in model evaluation, it is important to replicate the composite score in an independent validation cohort. Fourth, although our study covered a wide spectrum of proteins, it is a targeted proteomics study and may therefore miss changes in proteins that were unobserved in this study. In addition, the multiprotein composite score indicated that the current proteomics panel can discriminate MCI from control at high accuracy; however, the accuracy is lower in PTSD classification. It remains uncertain whether PTSD classification accuracy would be improved by surveying other proteins. Mass spectrometry is a competing platform for more comprehensive and hypothesis-free protein coverage. However, absent a targeted hypothesis, this platform requires a much larger sample size to rule out the greater numbers of false positives.

## Conclusion

To conclude, the current study identified several novel protein biomarkers for PTSD, MCI, and their co-occurrence. Many of these proteins have previously been implicated in other neurological and psychiatric disorders, in particular AD and schizophrenia. We also found substantial similarities in the profile of protein alterations of PTSD and MCI. This coincides with the evidence of shared heritability and molecular similarities across common brain disorders^51^. Our study further derived a multiprotein composite score that, upon replication and pending further refinement, could aid development of a practical, plasma-based assay to aid in classifying PTSD, MCI, and comorbid PTSD–MCI. Ultimately, the composite score could potentially be used to monitor patients longitudinally.

## Supplementary information

Supplementary Materials

Supplementary Figure 1

Supplementary Figure 2

Supplementary Table 1

Supplementary Table 2

Supplementary Table 3

Supplementary Table 4

Supplementary Table 6

## Data Availability

Proteomics data are included in Supplementary Table 1.

## References

[1] Bromet E (2016). DSM-IV post-traumatic stress disorder among World Trade Center responders 11–13 years after the disaster of 11 September 2001 (9/11). Psychol. Med..

[2] Cone JE (2015). Chronic probable PTSD in police responders in the world trade center health registry ten to eleven years after 9/11. Am. J. Ind. Med..

[3] Galea S (2002). Psychological sequelae of the September 11 terrorist attacks in New York City. N. Engl. J. Med..

[4] Kotov R (2015). Posttraumatic stress disorder and the risk of respiratory problems in World Trade Center responders: longitudinal test of a pathway. Psychosom. Med..

[5] Petersen RC (2011). Mild cognitive impairment. N. Engl. J. Med..

[6] Schuitevoerder S (2013). A meta-analysis of cognitive functioning in older adults with PTSD. J. Anxiety Disord..

[7] Rafferty LA, Cawkill PE, Stevelink SAM, Greenberg K, Greenberg N (2018). Dementia, post-traumatic stress disorder and major depressive disorder: a review of the mental health risk factors for dementia in the military veteran population. Psychol. Med..

[8] Clouston S (2017). Traumatic exposures, posttraumatic stress disorder, and cognitive functioning in World Trade Center responders. Alzheimers Dement..

[9] Nascimento JM, Martins-de-Souza D (2015). The proteome of schizophrenia. NPJ Schizophr..

[10] Cho WC (2007). Proteomics technologies and challenges. Genomics Proteom. Bioinforma..

[11] Crawford, M. E., Cusick, M. E. and Garrels, J. I. Databases and knowledge resources for proteomics research. *Trends Biotechnol*. **18**, 17–21 (2000).

[12] Kwon S-KC (2011). Stress and traumatic brain injury: a behavioral, proteomics, and histological study. Front. Neurol..

[13] Clouston SAP (2019). Posttraumatic stress disorder associated with total amyloid burden and amyloid-ß 42/40 ratios in plasma: results from a pilot study of World Trade Center responders. Alzheimer’s Dement..

[14] Rosen RL (2017). Elevated C-reactive protein and posttraumatic stress pathology among survivors of the 9/11 World Trade Center attacks. J. Psychiatr. Res..

[15] Shen, L. et al. Identifying neuroimaging and proteomic biomarkers for MCI and AD via the elastic net. In *Intl Workshop Multi. Brain Image Anal*. Springer, Berlin, Heidelberg, 27–34 (2011). 10.1007/978-3-642-24446-9_4 PMC4820290 27054198

[16] Agís‐Balboa RC (2017). Formin 2 links neuropsychiatric phenotypes at young age to an increased risk for dementia. EMBO J..

[17] Bayes A (2012). Comparative study of human and mouse postsynaptic proteomes finds high compositional conservation and abundance differences for key synaptic proteins. PLoS ONE.

[18] Neigh GN, Ali FF (2016). Co-morbidity of PTSD and immune system dysfunction: opportunities for treatment. Curr. Opin. Pharm..

[19] Schievink, S. H. J. et al. Cognitive changes in prevalent and incident cardiovascular disease: a 12-year follow-up in the Maastricht Aging Study (MAAS). *Eur. Heart J.* **0**, 1–8 (2017). 10.1093/eurheartj/ehx365 29020327

[20] Coughlin SS (2011). Post-traumatic stress disorder and cardiovascular disease. Open Cardiovasc. Med. J..

[21] Dasaro, C. R. et al. Cohort profile: world trade center health program general responder cohort. *Int. J. Epidemiol.* **46**, e9–e9 (2015). 10.1093/ije/dyv099 PMC6074831 26094072

[22] Miike K (2010). Proteome profiling reveals gender differences in the composition of human serum. Proteomics.

[23] Weathers, F. W., Litz, B. T., Herman, D. S., Huska, J. A. and Keane, T. M. The PTSD Checklist (PCL): reliability, validity, and diagnostic utility. In *Ann. Conv. Intl Soc. Traum. Stress Stud.*, San Antonio, TX, **462**, (1993).

[24] Nasreddine ZS (2005). The Montreal Cognitive Assessment, MoCA: a brief screening tool for mild cognitive impairment. J. Am. Geriatrics Soc..

[25] Albert MS (2011). The diagnosis of mild cognitive impairment due to Alzheimer’s disease: recommendations from the National Institute on Aging-Alzheimer’s Association workgroups on diagnostic guidelines for Alzheimer’s disease. Alzheimers Dement..

[26] Assarsson E (2014). Homogenous 96-plex PEA immunoassay exhibiting high sensitivity, specificity, and excellent scalability. PLoS ONE.

[27] Succop PA, Clark S, Chen M, Galke W (2004). Imputation of data values that are less than a detection limit. J. Occup. Environ. Hyg..

[28] Benjamini Y, Hochberg Y (1995). Controlling the false discovery rate: a practical and powerful approach to multiple testing. J. R. Stat. Soc. B.

[29] Zou H, Hastie T (2005). Regularization and variable selection via the elastic net. J. R. Stat. Soc.: Ser. B (Stat. Methodol.).

[30] Clouston SA (2016). Cognitive impairment among World Trade Center responders: long-term implications of re-experiencing the 9/11 terrorist attacks. Alzheimer’s Dement.: Diagnosis, Assess. Dis. Monit..

[31] Clouston SAP (2019). Incidence of mild cognitive impairment in World Trade Center responders: long-term consequences of re-experiencing the events on 9/11/2001. Alzheimers Dement..

[32] Clouston SAP (2019). Posttraumatic stress disorder and total amyloid burden and amyloid-beta 42/40 ratios in plasma: results from a pilot study of World Trade Center responders. Alzheimers Dement..

[33] Dean, K. R. et al. Multi-omic biomarker identification and validation for diagnosing warzone-related post-traumatic stress disorder. *Mol. Psychiatry* 1–13 (2019). 10.1038/s41380-019-0496-z PMC7714692 31501510

[34] Riese RJ (1998). Cathepsin S activity regulates antigen presentation and immunity. J. Clin. Investig..

[35] Grupe A (2007). Evidence for novel susceptibility genes for late-onset Alzheimer’s disease from a genome-wide association study of putative functional variants. Hum. Mol. Genet..

[36] Wu Z (2017). Cathepsin B plays a critical role in inducing Alzheimer’s disease-like phenotypes following chronic systemic exposure to lipopolysaccharide from *Porphyromonas gingivalis* in mice. Brain, Behav., Immun..

[37] Di Domenico, F., Tramutola, A. & Perluigi, M. *Cathepsin D as a Therapeutic Target in Alzheimer’s Disease* (Taylor & Francis, 2016). 10.1080/14728222.2016.1252334 27805462

[38] Bernstein HG, Bukowska A, Dobrowolny H, Bogerts B, Lendeckel U (2007). Cathepsin K and schizophrenia. Synapse.

[39] Mostafavi S (2018). A molecular network of the aging human brain provides insights into the pathology and cognitive decline of Alzheimer’s disease. Nat. Neurosci..

[40] Takeuchi A, O’Leary DD (2006). Radial migration of superficial layer cortical neurons controlled by novel Ig cell adhesion molecule MDGA1. J. Neurosci..

[41] Dines M, Lamprecht R (2016). The role of Ephs and Ephrins in memory formation. Int. J. Neuropsychopharmacol..

[42] Segman R (2005). Peripheral blood mononuclear cell gene expression profiles identify emergent post-traumatic stress disorder among trauma survivors. Mol. psychiatry.

[43] Breen MS (2018). PTSD blood transcriptome mega-analysis: shared inflammatory pathways across biological sex and modes of trauma. Neuropsychopharmacology.

[44] Hannan SB, Drager NM, Rasse TM, Voigt A, Jahn TR (2016). Cellular and molecular modifier pathways in tauopathies: the big picture from screening invertebrate models. J. Neurochem.

[45] Mehta D (2013). Childhood maltreatment is associated with distinct genomic and epigenetic profiles in posttraumatic stress disorder. Proc. Natl Acad. Sci. USA.

[46] Walker DG, Dalsing-Hernandez JE, Campbell NA, Lue LF (2009). Decreased expression of CD200 and CD200 receptor in Alzheimer’s disease: a potential mechanism leading to chronic inflammation. Exp. Neurol..

[47] Consortium C-DGotPG. (2013). Identification of risk loci with shared effects on five major psychiatric disorders: a genome-wide analysis. Lancet.

[48] Raum H (2015). A genome-wide supported psychiatric risk variant in NCAN influences brain function and cognitive performance in healthy subjects. Hum. Brain Mapp..

[49] DeWitt DA, Silver J, Canning DR, Perry G (1993). Chondroitin sulfate proteoglycans are associated with the lesions of Alzheimer’s disease. Exp. Neurol..

[50] Marx BP, Doron-Lamarca S, Proctor SP, Vasterling JJ (2009). The influence of pre-deployment neurocognitive functioning on post-deployment PTSD symptom outcomes among Iraq-deployed Army soldiers. J. Int. Neuropsychological Soc..

[51] Brainstorm C (2018). Analysis of shared heritability in common disorders of the brain. Science.

[52] Frenkel D (2013). Scara1 deficiency impairs clearance of soluble amyloid-β by mononuclear phagocytes and accelerates Alzheimer’s-like disease progression. Nat. Commun..

[53] Shyn SI (2011). Novel loci for major depression identified by genome-wide association study of sequenced treatment alternatives to relieve depression and meta-analysis of three studies. Mol. Psychiatry.

[54] Chen X (2017). A novel relationship for schizophrenia, bipolar, and major depressive disorder. Part 8: a hint from chromosome 8 high density association screen. Mol. Neurobiol..

[55] Kähler AK (2008). Association analysis of schizophrenia on 18 genes involved in neuronal migration: MDGA1 as a new susceptibility gene. Am. J. Med. Genet. Part B: Neuropsychiatr. Genet..

[56] Pettem KL, Yokomaku D, Takahashi H, Ge Y, Craig AM (2013). Interaction between autism-linked MDGAs and neuroligins suppresses inhibitory synapse development. J. Cell Biol..

[57] Solov’ev V, Gengin M (2007). Activity of peptidyl-dipeptidase a and carboxypeptidase N in the serum of patients with Alzheimer disease. Ukrains’ kyi biokhimichnyi Zh. (1999).

[58] Guilarte TR (2008). Dysregulation of glutamate carboxypeptidase II in psychiatric disease. Schizophrenia Res..

[59] Luedecking-Zimmer E, DeKosky ST, Chen Q, Barmada MM, Kamboh IM (2002). Investigation of oxidized LDL-receptor 1 (OLR1) as the candidate gene for Alzheimer’s disease on chromosome 12. Hum. Genet..

[60] Gareeva AE, Traks T, Koks S, Khusnutdinova EK (2015). The role of neurotrophins and neurexins genes in the risk of paranoid schizophrenia in Russians and Tatars. Genetika.

[61] Potvin S (2008). Inflammatory cytokine alterations in schizophrenia: a systematic quantitative review. Biol. Psychiatry.

[62] Toft H (2018). PTsD patients show increasing cytokine levels during treatment despite reduced psychological distress. Neuropsychiatr. Dis. Treat..

[63] Italiani P (2018). Circulating levels of IL-1 family cytokines and receptors in Alzheimer’s disease: new markers of disease progression?. J. Neuroinflammation.

[64] Wang T (2012). FGFR2 is associated with bipolar disorder: a large-scale case–control study of three psychiatric disorders in the Chinese Han population. World J. Biol. Psychiatry.

[65] O’Donovan MC (2009). Analysis of 10 independent samples provides evidence for association between schizophrenia and a SNP flanking fibroblast growth factor receptor 2. Mol. Psychiatry.

[66] Gonzalez-Mantilla AJ, Moreno-De-Luca A, Ledbetter DH, Martin CL (2016). A cross-disorder method to identify novel candidate genes for developmental brain disorders. JAMA Psychiatry.

[67] Suda S (2011). Decreased expression of axon-guidance receptors in the anterior cingulate cortex in autism. Mol. Autism.

[68] Beroun, A. et al. MMPs in learning and memory and neuropsychiatric disorders. *Cell. Mol. Life Sci.* **76**, 3207–3228 (2019). 10.1007/s00018-019-03180-8 PMC6647627 31172215

[69] Liu Y (2013). Matrix metalloproteinase-12 contributes to neuroinflammation in the aged brain. Neurobiol. Aging.

[70] Chopra K, Baveja A, Kuhad A (2015). MMPs: a novel drug target for schizophrenia. Expert Opin. Therapeutic Targets.

[71] Yazdani, A., Mendez-Giraldez, R., Kosorok, M. R. & Roussos, P. Transcriptomic causal networks identified patterns of differential gene regulation in human brain from Schizophrenia cases versus controls. Preprint at https://arxiv.org/abs/1908.07520 (2019). 10.1186/s12859-020-03753-6 PMC7579819 33087039

[72] Fuchs A, Cella M, Giurisato E, Shaw AS, Colonna M (2004). Cutting edge: CD96 (tactile) promotes NK cell-target cell adhesion by interacting with the poliovirus receptor (CD155). J. Immunol..

[73] Shao L, Vawter MP (2008). Shared gene expression alterations in schizophrenia and bipolar disorder. Biol. Psychiatry.

[74] Han Z, Huang H, Gao Y, Huang Q (2017). Functional annotation of Alzheimer’s disease associated loci revealed by GWASs. PLoS ONE.

[75] Cruchaga C (2010). SNPs associated with cerebrospinal fluid phospho-tau levels influence rate of decline in Alzheimer’s disease. PLoS Genet..

[76] Kuester M, Kemmerzehl S, Dahms SO, Roeser D, Than ME (2011). The crystal structure of death receptor 6 (DR6): a potential receptor of the amyloid precursor protein (APP). J. Mol. Biol..

[77] Nakamura Y (1991). Abnormal distribution of cathepsins in the brain of patients with Alzheimer’s disease. Neurosci. Lett..

[78] Lendeckel U (2009). Cathepsin K generates enkephalin from beta-endorphin: a new mechanism with possible relevance for schizophrenia. Neurochem. Int..

[79] Narayanan B (2015). Multivariate genetic determinants of EEG oscillations in schizophrenia and psychotic bipolar disorder from the BSNIP study. Transl. Psychiatry.

[80] Chen K-L (2009). The epigenetic effects of amyloid-β1–40 on global DNA and neprilysin genes in murine cerebral endothelial cells. Biochem. Biophys. Res. Commun..

[81] Roig B (2007). The discoidin domain receptor 1 as a novel susceptibility gene for schizophrenia. Mol. Psychiatry.

[82] Jang M (2013). Secreted frizzled-related protein 3 (sFRP3) regulates antidepressant responses in mice and humans. Mol. Psychiatry.

[83] Weeraratna AT (2007). Alterations in immunological and neurological gene expression patterns in Alzheimer’s disease tissues. Exp. Cell Res..

[84] Di Zenzo G (2012). Pemphigus autoantibodies generated through somatic mutations target the desmoglein-3 cis-interface. J. Clin. Investig..

[85] Snijders C (2020). Longitudinal epigenome-wide association studies of three male military cohorts reveal multiple CpG sites associated with post-traumatic stress disorder. Clin. Epigenetics.

[86] Redies C, Hertel N, Hubner CA (2012). Cadherins and neuropsychiatric disorders. Brain Res..

[87] Whelan CD (2019). Multiplex proteomics identifies novel CSF and plasma biomarkers of early Alzheimer’s disease. Acta Neuropathol. Commun..

[88] Niculescu, A. et al. Blood biomarkers for memory: toward early detection of risk for Alzheimer disease, pharmacogenomics, and repurposed drugs. *Mol. Psychiatry* **25**, 1651–1672 (2020). 10.1038/s41380-019-0602-2 PMC7387316 31792364

[89] Srinivasan, S. et al. Studying the epigenetic regulations of posttraumatic stress disorder (PTSD) using a social defeat mouse model. *FASEB J.* **25**, 507-509 (2011).

[90] Goswami DB (2013). Gene expression analysis of novel genes in the prefrontal cortex of major depressive disorder subjects. Prog. Neuropsychopharmacol. Biol. Psychiatry.

[91] Misiak B, Stramecki F, Stanczykiewicz B, Frydecka D, Lubeiro A (2018). Vascular endothelial growth factor in patients with schizophrenia: a systematic review and meta-analysis. Prog. Neuropsychopharmacol. Biol. Psychiatry.

[92] Hohman TJ, Bell SP, Jefferson AL, Alzheimer’s Disease, Neuroimaging I (2015). The role of vascular endothelial growth factor in neurodegeneration and cognitive decline: exploring interactions with biomarkers of Alzheimer disease. JAMA Neurol..

[93] Kong W (2009). Independent component analysis of Alzheimer’s DNA microarray gene expression data. Mol. Neurodegener..

[94] Martins-de-Souza D (2009). Prefrontal cortex shotgun proteome analysis reveals altered calcium homeostasis and immune system imbalance in schizophrenia. Eur. Arch. Psychiatry Clin. Neurosci..

[95] Xie P (2013). Genome-wide association study identifies new susceptibility loci for posttraumatic stress disorder. Biol. Psychiatry.

[96] Sahin P, McCaig C, Jeevahan J, Murray JT, Hainsworth AH (2013). The cell survival kinase SGK1 and its targets FOXO3a and NDRG1 in aged human brain. Neuropathol. Appl. Neurobiol..

[97] Miyata S, Hattori T, Shimizu S, Ito A, Tohyama M (2015). Disturbance of oligodendrocyte function plays a key role in the pathogenesis of schizophrenia and major depressive disorder. Biomed. Res. Int..

[98] Kam TI (2013). FcgammaRIIb mediates amyloid-beta neurotoxicity and memory impairment in Alzheimer’s disease. J. Clin. Invest..

[99] Breen MS (2015). Gene networks specific for innate immunity define post-traumatic stress disorder. Mol. Psychiatry.

[100] Rentzos M (2006). IL-15 is elevated in cerebrospinal fluid of patients with Alzheimer’s disease and frontotemporal dementia. J. Geriatr. Psychiatry Neurol..

