# Supplementary material for: Molecular linkage between post-traumatic stress disorder and cognitive impairment: a targeted proteomics study of World Trade Center responders
Source: Transl Psychiatry. 2020 Aug 4;10:269. doi: 10.1038/s41398-020-00958-4 (PMC7403297; doi:10.1038/s41398-020-00958-4)
Supplement: Supplementary file 1 — Supplementary Materials [file 41398_2020_958_MOESM1_ESM.docx]

**Supplementary Materials**

*Comparison of results to recent omics studies*

We compared our top proteins to recent omics studies of PTSD and Alzheimer’s disease (AD) ^1-8^ summarized below.

1. In the gene expression study of Mostafavi, Gaiteri ^8^, the authors computed the Spearman rank correlation (for continuous trait) and t-test (for binary trait) to assess the association between the expression level of each gene and 5 AD-related traits, namely pathologic diagnosis of AD, clinical diagnosis of AD, β-amyloid load, tau tangle density and cognitive decline. The authors provided the list of signed negative log p-values in Supplementary Table 2 of their paper. There were 13483 rows in this table, however some genes were duplicated. After removing duplicated gene symbols, 13467 unique genes remained. For each AD-related trait, we converted the values back to p-values and computed FDR. 99, 856, 2316, 241 and 3036 genes were significantly associated with pathologic diagnosis of AD, clinical diagnosis of AD, β-amyloid load, tau tangle density and cognitive decline, respectively at FDR < 0.05. 3/99 genes from pathologic diagnosis of AD analysis overlapped with our panel of 276 proteins. MDGA1 was significant at p < 0.05 in PTSD-MCI and MCI-only analysis. 7/856 genes from clinical diagnosis of AD analysis overlapped with our panel of 276 proteins. EPHA10 was significant at p < 0.05 in PTSD-MCI and MCI-only analysis. 24/2316 genes from β-amyloid load analysis overlapped with our panel of 276 proteins. CD200, DDR1, EPHA10, FGFR2, MDGA1, NDRG1 and NXPH1 were significant at p < 0.05 in our analysis, however the effect sizes for CD200 and NDRG1 were in opposite directions compared to Mostafavi, Gaiteri ^8^. For tau tangle density analysis, none of the 241 genes overlapped with our panel of 276 proteins. 27/3036 genes from cognitive decline analysis overlapped with our panel of 276 proteins. CD200, CD302, CTF1, EPHA10, FGFR2, MDGA1, NXPH1 and VWC2 were significant at p < 0.05 in our analysis, however the effect sizes of CD200, CTF1 and VWC2 were in opposite directions compared to Mostafavi, Gaiteri ^8^. The complete list of overlapping genes was provided in Supplementary Table 2. On the other hand, using p < 0.05 as threshold, 2056, 2969, 4286, 2482, 4752 genes were significantly associated with pathologic diagnosis of AD, clinical diagnosis of AD, β-amyloid load, tau tangle density and cognitive decline, respectively. The comparison based on overlapping genes at p < 0.05 from Mostafavi, Gaiteri ^8^ was provided in Supplementary Table 3.
2. In the gene expression study of Breen, Tylee ^5^, the authors performed a mega-analysis of 5 studies in PTSD related to combat and interpersonal trauma ^9-13^. Using the list of genes at p < 0.05 for each study (which were further divided into combat and interpersonal trauma sub cohorts) provided in Supplementary 2 of Breen, Tylee ^5^, there were 951, 584, 485, 309, 294, 427 and 157 genes associated with Mehta, Klengel ^10^ childhood trauma, Neylan, Sun ^11^ assault trauma, Segman, Shefi ^12^ emergency room trauma, Mehta, Klengel ^10^ interpersonal trauma, Neylan, Sun ^11^ combat trauma, Breen, Maihofer ^9^ combat trauma and Tylee, Chandler ^13^ combat trauma, respectively. 9/951 genes from Mehta, Klengel ^10^ childhood trauma overlapped with our panel of 276 proteins. CD200 was significant at p < 0.05 in PTSD-only and MCI-only analysis. 6/584 genes from Neylan, Sun ^11^ assault trauma overlapped with our panel of 276 proteins, none was significant at p < 0.05 in our study. 7/485 genes from Segman, Shefi ^12^ overlapped with our panel of 276 proteins. ATP6V1F was significant at p < 0.05 in PTSD-only and MCI-only analysis, whereas MSR1 was significant at FDR < 0.1 in PTSD-MCI analysis. 7/309 genes from Mehta, Klengel ^10^ interpersonal trauma overlapped with our panel of 276 proteins. CD200 was significant at p < 0.05 in PTSD-only and MCI-only analysis. LXN was significant at p < 0.05 in MCI-only analysis. 3/294 genes from Neylan, Sun ^11^ combat trauma overlapped with our panel of 276 proteins. TNFRSF21 was significant at p < 0.05 in PTSD-only analysis, however the effect size was in opposite direction compared to Neylan, Sun ^11^. 6/427 genes from Breen, Maihofer ^9^ combat trauma overlapped with our panel of 276 proteins, none was significant at p < 0.05 in our study. Finally none of the 157 genes from Tylee, Chandler ^13^ combat trauma overlapped with our panel of 276 proteins. The complete list of overlapping genes was provided in Supplementary Table 4.
3. In the GWAS of Gelernter, Sun ^1^, the authors identified 8 loci associated with reexperiencing symptoms among European American in US veterans. All these 8 loci mapped to intronic region of closest gene. Only MAD1L1 overlapped with our panel of 276 proteins. MAD1L1 was not significantly associated with PTSD-MCI, PTSD-only or MCI-only in our study.
4. In the gene expression study of Rusch, Robinson ^2^, the authors identified 98 probesets, representing 89 distinct genes and 4 uncharacterized genes to be DE between PTSD (n = 39) compared to controls (n = 27). Among these 89 genes, only LEPR overlapped with our panel of 276 proteins. LEPR was not statistically significantly associated with PTSD-MCI, PTSD-only or MCI-only in our study, although the effect size was positive (consistent with upregulation of LEPR in Rusch, Robinson ^2^).
5. In the GWAS of Kunkle, Grenier-Boley ^3^, the authors identified 25 loci associated with late onset AD in meta-analysis of non-Hispanic Whites. None of the genes closest to these loci overlapped with our panel of 276 proteins.
6. In the gene expression study of Mehta, Voisey ^4^, the authors identified 60 genes at Bonferroni p < 0.05 comparing veterans with PTSD (n = 48) and without PTSD (n = 48). Among these 60 genes, only LEP and TGM2 overlapped with our panel of 276 proteins. These two genes were not statistically significantly associated with PTSD-MCI, PTSD-only or MCI-only in our study.
7. In the review paper of Girgenti and Duman ^6^ on GWAS and gene expression for PTSD, we extracted the genes mentioned in the paper to be associated with PTSD (10 genes). FKBP5, IL16 and IL18 overlapped with our panel of 276 proteins, however none was significantly associated with PTSD-MCI, PTSD-only and MCI-only in our study.
8. In the GWAS of Jun, Chung ^7^, the authors performed a meta-analysis for AD. We extracted the genes mapping to loci identified by Jun, Chung ^7^ associated with AD in European American from SNP and SNP × APOE interaction tests, as well as loci identified from transethic analyses reported in the paper (13 genes altogether). None of these genes overlapped with our panel of 276 proteins.
9. In the GWAS of Lambert, Ibrahim-Verbaas ^14^, the authors identified 19 loci associated with AD. None of these genes overlapped with our panel of 276 proteins.

**References**

1. Gelernter J *et al.* Genome-wide association study of post-traumatic stress disorder reexperiencing symptoms in >165,000 US veterans. *Nat Neurosci* 2019; **22**(9)**:** 1394-1401.

2. Rusch HL *et al.* Gene expression differences in PTSD are uniquely related to the intrusion symptom cluster: A transcriptome-wide analysis in military service members. *Brain Behav Immun* 2019; **80:** 904-908.

3. Kunkle BW *et al.* Genetic meta-analysis of diagnosed Alzheimer's disease identifies new risk loci and implicates Abeta, tau, immunity and lipid processing. 2019; **51**(3)**:** 414-430.

4. Mehta D *et al.* Transcriptome analysis reveals novel genes and immune networks dysregulated in veterans with PTSD. *Brain Behav Immun* 2018; **74:** 133-142.

5. Breen MS *et al.* PTSD Blood Transcriptome Mega-Analysis: Shared Inflammatory Pathways across Biological Sex and Modes of Trauma. *Neuropsychopharmacology* 2018; **43**(3)**:** 469-481.

6. Girgenti MJ, Duman RS. Transcriptome Alterations in Posttraumatic Stress Disorder. *Biol Psychiatry* 2018; **83**(10)**:** 840-848.

7. Jun GR *et al.* Transethnic genome-wide scan identifies novel Alzheimer's disease loci. *Alzheimers Dement* 2017; **13**(7)**:** 727-738.

8. Mostafavi S *et al.* A molecular network of the aging human brain provides insights into the pathology and cognitive decline of Alzheimer's disease. *Nat Neurosci* 2018; **21**(6)**:** 811-819.

9. Breen MS *et al.* Gene networks specific for innate immunity define post-traumatic stress disorder. *Mol Psychiatry* 2015; **20**(12)**:** 1538-1545.

10. Mehta D *et al.* Childhood maltreatment is associated with distinct genomic and epigenetic profiles in posttraumatic stress disorder. *Proceedings of the National Academy of Sciences* 2013; **110**(20)**:** 8302-8307.

11. Neylan TC *et al.* Suppressed monocyte gene expression profile in men versus women with PTSD. *Brain Behav Immun* 2011; **25**(3)**:** 524-531.

12. Segman R *et al.* Peripheral blood mononuclear cell gene expression profiles identify emergent post-traumatic stress disorder among trauma survivors. *Molecular psychiatry* 2005; **10**(5)**:** 500-513.

13. Tylee DS *et al.* Blood-based gene-expression biomarkers of post-traumatic stress disorder among deployed marines: A pilot study. *Psychoneuroendocrinology* 2015; **51:** 472-494.

14. Lambert JC *et al.* Meta-analysis of 74,046 individuals identifies 11 new susceptibility loci for Alzheimer's disease. *Nat Genet* 2013; **45**(12)**:** 1452-1458.

**Supplementary Figure Legends**

**Supplementary Figure 1.** Reproducibility of Olink assay across 6 sets of bridging samples (i.e., technical duplicates) (average r=0.97).

**Supplementary Figure 2.** A-F. Control versus PTSD-only versus PTSD-CI subgroups for A. NCAN, B. BCAN, C. CTSS, D. MSR1, E. MDGA1, F. CPA2. G-L. Control versus MCI-only versus PTSD-MCI subgroups for G. NCAN, H. BCAN, I. CTSS, J. MSR1, K. MDGA1, L. CPA2. Dashed purple box implied that the two subgroups were similar to each other based on BIC scores.

**Supplementary Table 1 Legend.** Proteomics dataset.

**Supplementary Table 2 Legend.** Comparison of overlapping genes identified at FDR < 0.05 from Mostafavi, Gaiteri ^8^. For cognitive decline, effect size direction was consistent if the association in Mostafavi, Gaiteri ^8^ was in opposite sign compared to our case/control since MCI (case) was associated with lower cognitive decline score. ** and * implied the gene was significant at FDR < 0.01 and p < 0.05 in our study, respectively. ns: not significant.

**Supplementary Table 3 Legend.** Comparison of overlapping genes identified at p < 0.05 from Mostafavi, Gaiteri ^8^. For cognitive decline, effect size direction was consistent if association in Mostafavi, Gaiteri ^8^ was in opposite sign compared to our case/control since MCI (case) was associated with lower cognitive decline score. ** and * implied the gene was significant at FDR < 0.01 and p < 0.05 in our study, respectively. ns: not significant.

**Supplementary Table 4 Legend.** Comparison of overlapping genes identified at p < 0.05 from Breen, Tylee ^5^ for 5 PTSD studies (which were further divided into combat and interpersonal trauma sub cohorts). ** and * implied the gene was significant at FDR < 0.01 and p < 0.05 in our study, respectively. ns: not significant.

**Supplementary Table 5.** BIC scores computed from the models comparing models *H1-H3* for (a) Control versus PTSD-only versus PTSD-MCI and (b) Control versus MCI-only versus PTSD-MCI.

| 1. Control versus PTSD-only versus PTSD-MCI | | | | | | | | |
| --- | --- | --- | --- | --- | --- | --- | --- | --- |
|  | *H1*: Control < PTSD-only < PTSD-MCI or Control > PTSD-only > PTSD-MCI | *H2*: Control ≠ PTSD-only = PTSD-MCI | *H3*: Control =PTSD-only ≠ PTSD-MCI | | Best Model | |  |  |
| NCAN | -649.06 | -641.4 | | -649.08 | | H3 | |  |
| CPA2 | -444.67 | -442.11 | | -443.53 | | H1 | |  |
| MSR1 | -453.02 | -451.2 | | -449.78 | | H1 | |  |
| BCAN | -443.24 | -440.75 | | -439.63 | | H1 | |  |
| MDGA1 | -237 | -235.75 | | -233.75 | | H1 | |  |
| CTSS | -715.99 | -715.2 | | -711.5 | | H1 | |  |
| 1. Control versus MCI-only versus PTSD-MCI | | | | | | | | |
|  | *H1*: Control < MCI-only < PTSD-MCI or Control > MCI-only > PTSD-&MCI | *H2*: Control ≠ MCI-only = PTSD-MCI | | *H3*: Control = MCI-only ≠ PTSD-MCI | | Best Model | |  |
| NCAN | -578.88 | -575.25 | | -576.01 | | H1 | |  |
| CPA2 | -420.90 | -422.33 | | -415.90 | | H2 | |  |
| MSR1 | -392.28 | -389.29 | | -393.45 | | H3 | |  |
| BCAN | -408.36 | -409.56 | | -402.27 | | H2 | |  |
| MDGA1 | -222.80 | -223.40 | | -218.23 | | H2 | |  |
| CTSS | -636.76 | -637.18 | | -632.65 | | H2 | |  |

**Supplementary Table 6 Legend.** List of proteins differentially expressed at p < 0.1 from the subset analyses.

**Supplementary Table 7.** Additional leave-one-out cross-validation predictions performance on models trained on subsets of (a) PTSD-MCI, (b) PTSD-only and (c) MCI-only versus controls.

| Classification | Candidate feature set | AUC | Correlation with PCL | Correlation with MoCA score |
| --- | --- | --- | --- | --- |
| PTSD-MCI *versus* control | 16 PTSD-MCI associated Olink proteins from Table 2 | 0.81 | 0.47 (p < 0.001) | -0.48 (p < 0.001) |
|  | All 276 proteins | 0.70 | 0.37 (p < 0.001) | -0.34 (p < 0.001) |
| PTSD-only *versus* control | 24 PTSD-only associated proteins from Table 2 | 0.68 | 0.25 (p = 0.006) | -0.021 (p = 0.83) |
|  | All 276 proteins | 0.56 | 0.03 (p = 0.74) | -0.10 (p = 0.30) |
| MCI-only *versus* control | 20 MCI-only associated proteins from Table 2 | 0.84 | 0.26 (p = 0.008) | -0.57 (p < 0.001) |
|  | All 276 proteins | 0.56 | 0.22 (p = 0.021) | -0.16 (p = 0.080) |
