# Supplementary figures and images for: Molecular linkage between post-traumatic stress disorder and cognitive impairment: a targeted proteomics study of World Trade Center responders
Source: Transl Psychiatry. 2020 Aug 4;10:269. doi: 10.1038/s41398-020-00958-4 (PMC7403297; doi:10.1038/s41398-020-00958-4)

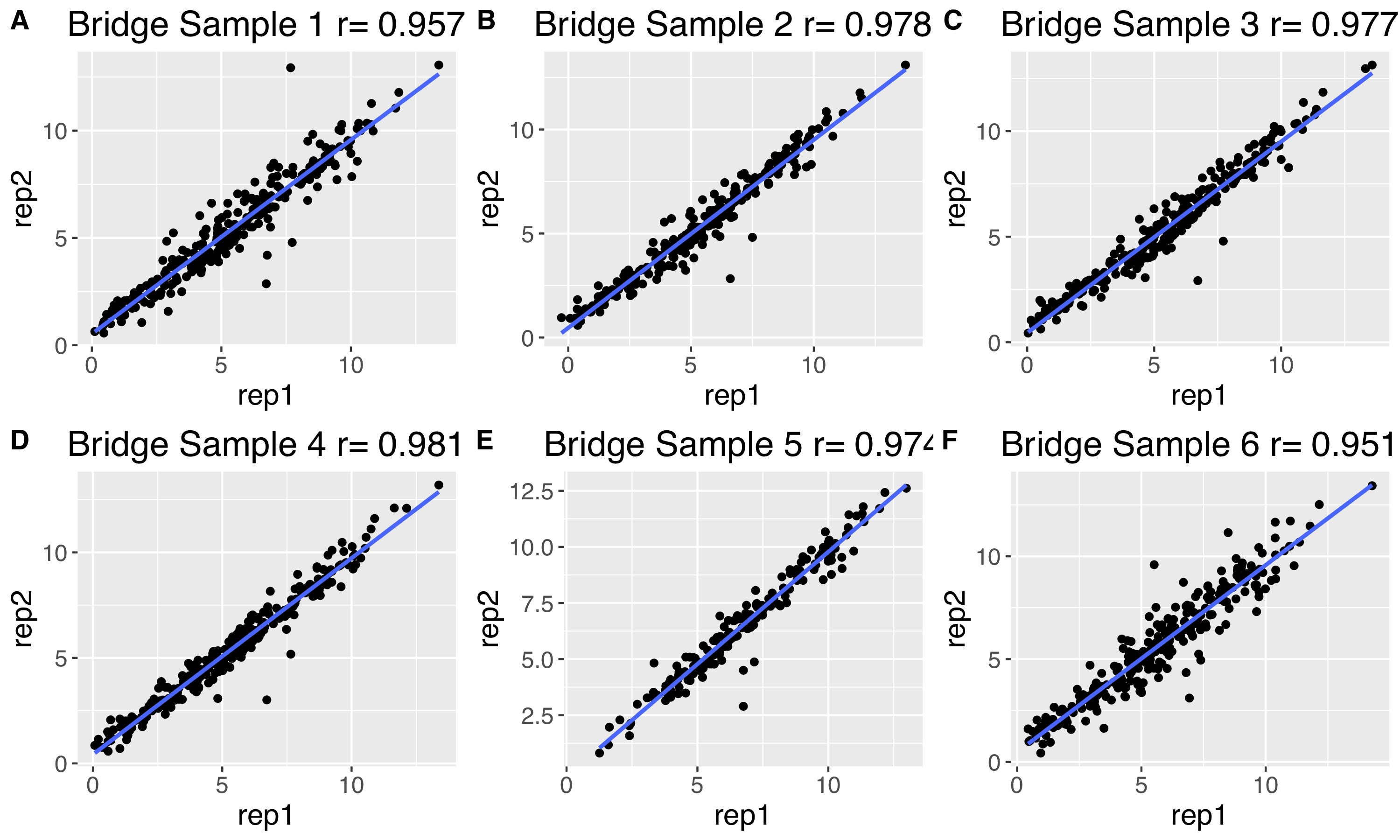

Supplement: Supplementary file 2 — Supplementary Figure 1 [file 41398_2020_958_MOESM2_ESM.jpg]

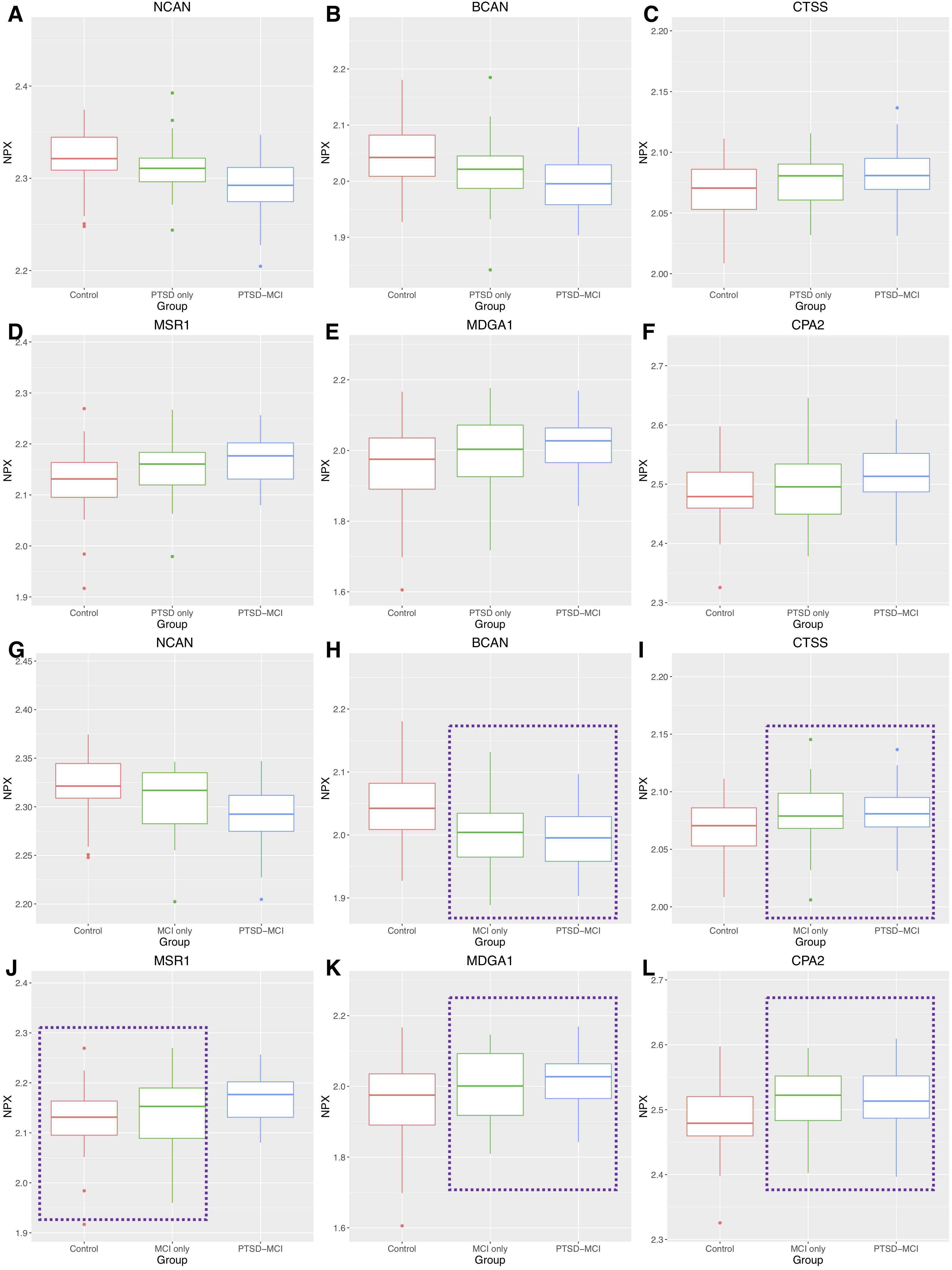

Supplement: Supplementary file 3 — Supplementary Figure 2 [file 41398_2020_958_MOESM3_ESM.jpg]
